# Expectations guide predictive eye movements and information sampling during face recognition

**DOI:** 10.1016/j.isci.2024.110920

**Published:** 2024-09-10

**Authors:** Annika Garlichs, Mark Lustig, Matthias Gamer, Helen Blank

**Affiliations:** 1 Department of Systems Neuroscience, University Medical Center Hamburg-Eppendorf, Hamburg, Germany; 2 Hamburg Brain School, University Medical Center Hamburg-Eppendorf, Hamburg, Germany; 3 Department of Psychology, University of Hamburg, Hamburg, Germany; 4 Department of Psychology, University of Würzburg, Würzburg, Germany; 5 Predictive Cognition, Research Center One Health Ruhr of the University Alliance Ruhr, Faculty of Psychology, Ruhr-University Bochum, Bochum, Germany

**Keywords:** Biological sciences, Cognitive neuroscience, Social sciences, Research methodology social sciences

## Abstract

Context information has a crucial impact on our ability to recognize faces. Theoretical frameworks of predictive processing suggest that predictions derived from context guide sampling of sensory evidence at informative locations. However, it is unclear how expectations influence visual information sampling during face perception. To investigate the effects of expectations on eye movements during face anticipation and recognition, we conducted two eye-tracking experiments (*n* = 34, each) using cued face morphs containing expected and unexpected facial features, and clear expected and unexpected faces. Participants performed predictive saccades toward expected facial features and fixated expected more often and longer than unexpected features. In face morphs, expected features attracted early eye movements, followed by unexpected features, indicating that top-down as well as bottom-up information drives face sampling. Our results provide compelling evidence that expectations influence face processing by guiding predictive and early eye movements toward anticipated informative locations, supporting predictive processing.

## Highlights

• Predictive coding suggests an active sampling of expected information
• We studied visual information sampling during face perception
• Participants performed predictive saccades and early fixations of expected features
• Expectations guide eye movements toward locations of interest

## Graphical abstract

**Figure:**
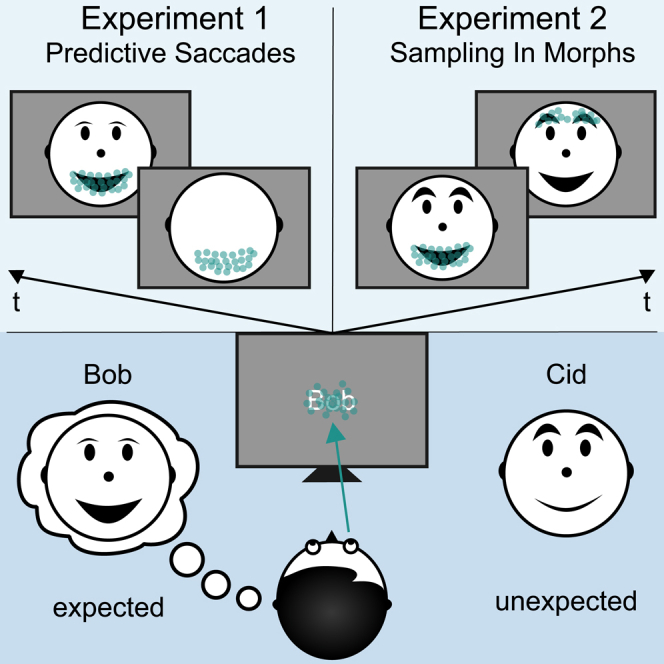

## Introduction

Context provides important information for recognizing faces as one of the most important stimuli in everyday life.^1^^,^^2^^,^^3^ While behavioral evidence indicates that expectations can facilitate face recognition, especially when the face is degraded or ambiguous,^4^^,^^5^^,^^6^^,^^7^ it is unclear whether context already guides how visual information is sampled when we look at a face. Frameworks of predictive processing suggest an active sampling of informative locations, guided by prior beliefs.^8^^,^^9^ This process may direct eye movements toward expected sources of sensory input, aiming to reduce uncertainty in predictions.^10^

Context efficiently influences gazing behavior in complex everyday scenes, making one faster at locating a pot than a printer in a kitchen due to semantic and episodic knowledge about typical occurrences of objects^11^^,^^12^^,^^13^^,^^14^; for review, see Wolfe.^15^ Expectations can also guide predictive saccades ahead of time to anticipated regions of interest, e.g., toward ball locations during fast-paced games^16^^,^^17^^,^^18^ or prospective steps when walking down a staircase.^19^ While examining objects or scenes, expectations can lead to an earlier and increased sampling of either expected information^14^^,^^20^ or unexpected information.^21^^,^^22^

Previous eye-tracking studies on face perception revealed that humans typically first look at a point slightly below the eyes,^23^^,^^24^ before fixating on the eyes and mouth,^25^^,^^26^ areas specifically relevant for emotion recognition.^27^^,^^28^ Crucially, gaze patterns to faces can be modulated by context such as emotional content (for review, see Aviezer et al.^29^), familiarity,^30^^,^^31^^,^^32^^,^^33^ or ethnicity of the anticipated face.^34^ For instance, fixation patterns for angry faces are altered if the face is shown in a disgust context (e.g., the person is holding a trash bag), counterbalancing the typical bias toward the eye region. Conversely, if a disgusted face is shown in an anger context (e.g., the person is showing a fist), the usually symmetrical scanning pattern to the mouth and eye regions is shifted toward the eyes, which is already evident at the first fixation.^35^ Effects of familiarity on viewing behavior during face identity recognition are mixed. In some studies, there were more fixations toward the eye region for novel or unfamiliar faces, a location allowing holistic face processing, whereas famous or personally familiar faces had overall reduced upper-face fixations.^31^^,^^33^ In contrast, other studies using name recall and fame judgment instead of familiarity judgment tasks reported an increased sampling of the eye region for familiar or famous compared to unfamiliar faces.^30^^,^^32^ Gaze patterns toward facial features also vary depending on whether viewers anticipate a displayed face morph to be Chinese or Caucasian, directing the gaze toward either the nose or the eyes, respectively.^34^ Taken together, these findings suggest that expectations derived from surrounding emotional context, familiarity, or general knowledge about racial categories influence how people look at face images.

**Figure 1:**
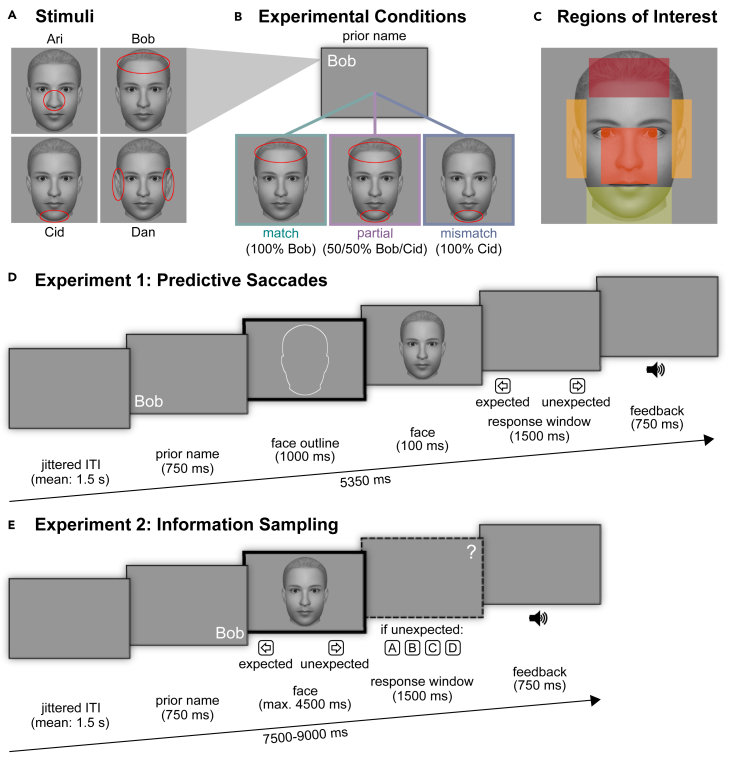
Experimental procedure (A) Stimuli: Images of four identities were used. Each identity had one distinct facial feature (here shown with a red circle). (B) Experimental conditions: in both experiments, a name prior was depicted randomly in one of the four corners. Afterward, a face was presented. The face could be expected (*match*), unexpected (*mismatch*), or a morph containing the expected as well as an unexpected identity (*partial*; 50/50% morphs). (C) Regions of interest (ROIs): We used four ROIs of identical size that covered the distinct features of the four identities. (D) Exemplary trial of Experiment 1: after the presentation of a name prior, an outline of the “base face” was presented. Next, a face was shown for 100 ms. The task for the participant was to indicate whether the face had been “expected’ or “unexpected” based on the preceding name. Participants received auditory feedback on whether they answered correctly, incorrectly, or too slowly. The thick black frame indicates the time window for our eye-tracking analyses. ITI, inter-trial interval (jittered between 1.25 and 1.75 s, mean 1.5 s). (E) Exemplary trial of Experiment 2: in contrast to Experiment 1, the face was presented up to 4,500 ms or until a button press. The first task for the participants was to indicate whether the presented face was “expected” or “unexpected” based on the preceding name. If participants answered “unexpected,” participants were required to answer with one out of four buttons which identity they saw in the presented face. Auditory feedback (too slow) was provided.

We investigated how expectations about an upcoming face with particular facial features affect eye movements during anticipation and perception of that face in two preregistered experiments. Participants learned to associate images of four male faces with names in both experiments. Each face was characterized by one distinct facial feature, such as a high forehead or a wide chin. In each trial, expectations about the upcoming face and hence its distinct facial feature were induced by a name prior. Furthermore, we showed face morphs between two identities, containing the expected and an unexpected identity. In Experiment 1, we hypothesized that participants would use context information to perform predictive saccades toward locations containing the expected facial feature. To enhance the use of predictive information, we inserted a long time interval before image presentation and provided only limited sensory information by restricting stimulus duration (Figure 1D). In Experiment 2, we investigated whether context information influences how participants sample face information. We hypothesized that expectations modulate active sampling, such that locations associated with the expected identity are preferentially sampled, evident by (1) an initial fixation at the location of the expected facial feature in all faces as well as (2) more fixations and longer dwell time on the expected feature in face morphs, containing expectation-compliant as well as -incompliant information (Figures 1B and 1E).

## Results

### Facilitation and assimilation effect due to expectation

**Figure 2:**
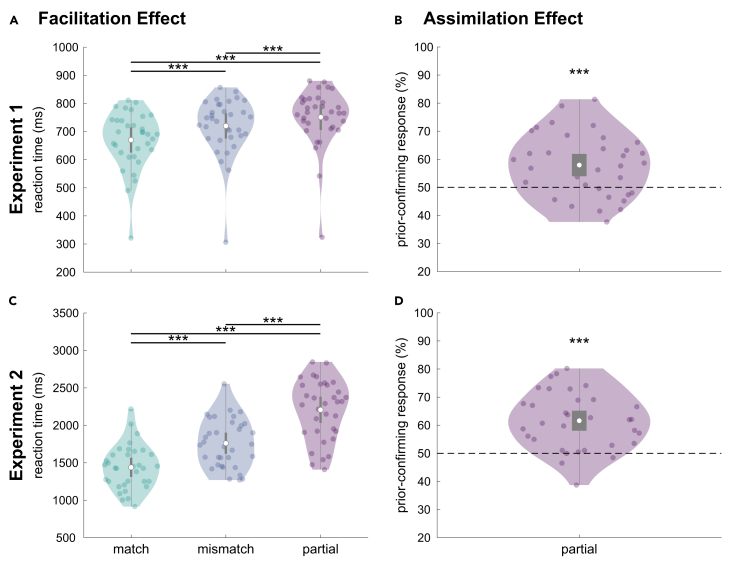
Behavioral results of experiments 1 and 2 (A and C) Facilitation effect: participants reacted faster to expected (*match* condition) compared to unexpected (*mismatch* condition) or morphed (*partial* condition) faces. Dots represent single participants. The white dots represent means, gray rectangles 95% confidence intervals, and the lower and upper whiskers Q_1/3_ −/+ 1.5∗interquartile range. In (A) and (C), black lines indicate *p* < 0.001. (B and D) Assimilation effect: participants responded more often to have perceived the expected identity in a face morph (partial condition). The dashed line represents the chance level, i.e., perceiving the expected or unexpected identity equally often. Asterisks indicate *p* < 0.001. The upper and lower rows depict the results for experiments 1 and 2, respectively.

**Figure 3:**
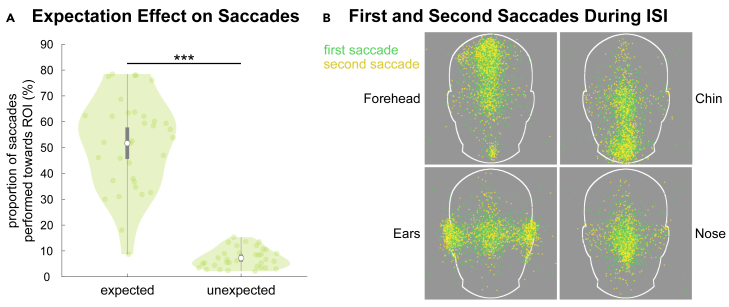
Eye-tracking results of Experiment 1 (A) Expectation-induced predictive saccades: participants performed saccades more often toward a region of interest (ROI) if the corresponding facial feature had been expected compared to when it had not been expected (main effect “expectation”). Each dot displays, for each participant, the average percentage of fixations toward an ROI if its facial feature has been expected or not (averaged across the four ROIs). The white dots represent means, gray rectangles 95% confidence intervals, and the lower and upper whiskers Q_1/3_ −/+ 1.5∗interquartile range. The black line indicates *p* < 0.001. (B) Visualization of predictive saccades: predictive saccades clustered depending on where the distinct facial feature was expected (forehead, chin, ears, and nose). Single dots represent the endpoints of all participants’ first and second saccades during the presentation of the face outline in the inter-stimulus interval (ISI) between the name prior and a presented face. Green, first saccade; yellow, second saccade.

In both experiments, prior expectations induced a facilitation effect, i.e., faster classification of expected compared to (partially) unexpected faces as evident by the main effects of “condition” (*match*, *mismatch*, *partial*) on reaction times (RTs) (Experiment 1: *F*(1.97, 65.09) = 77.72, *p* < 0.001, *η*^*2*^ = 0.10; Experiment 2: *F*(1.43, 47.15) = 182.40, *p* < 0.001, *η*^*2*^ = 0.47; Figures 2A and 2C). Specifically, participants were faster in classifying a clear face as expected or unexpected if the face matched their expectation compared to when it did not match their expectation at all (*match* vs. *mismatch*: Experiment 1: *match*: *M* = 670.07 ms, *SD* = 101.16 ms; *mismatch*: *M* = 719.86 ms, *SD* = 102.22 ms; *t*(33) = −8.05, *p* < 0.001, 95% confidence interval [CI] [−65.40, −34.20], *d* = 1.38, Figure 2A; Experiment 2: *match*: *M* = 1439.10 ms, *SD* = 290.96 ms; *mismatch*: *M* = 1761.00 ms, *SD* = 321.00 ms; *t*(33) = −12.19, *p* < 0.001, 95% CI [−389, −255], *d* = 2.09, Figure 2C). Similarly, expected faces were classified faster than face morphs containing the expected as well as an unexpected facial feature (*match* vs. *partial*: Experiment 1: *partial*: *M* = 751.16 ms, *SD* = 103.96 ms; *t*(33) = −11.84, *p* < 0.001, 95% CI [−98.40, −63.80], *d* = 2.03; Experiment 2: *partial*: *M* = 2207.50 ms, *SD* = 398.45 ms; *t*(33) = −15.43, *p* < 0.001, 95% CI [−894, −643], *d* = 2.65). Lastly, unexpected faces that completely mismatched expectations were classified faster than face morphs that contained information of two identities (*mismatch* vs. *partial*: Experiment 1: *t*(33) = −4.72, *p* < 0.001, 95% CI [−48.00, −14.60], *d* = 0.81, Figure 2A; Experiment 2: *t*(33) = −10.76, *p* < 0.001, 95% CI [−551, −342], *d* = 1.85, Figure 3A).

Prior expectations led to an assimilation effect in both experiments; i.e., face morphs were more often classified as the expected identity (Experiment 1: *t*(33) = 4.12, *p* < 0.001, 95% CI [54.02, 61.85], *d* = 0.71, Figure 2B; Experiment 2: *t*(33) = 6.75, *p* < 0.001, 95% CI [58.11, 65.10], *d* = 1.16, Figure 2D).

In addition, participants performed well in identifying the clear faces in both experiments. The presented face was correctly identified in the *match* (Experiment 1: *M* = 86.43%, *SD* = 9.84%, Z = 5.08, *p* < 0.001, Wilcoxon’s *r* = 0.87; Experiment 2: *M* = 98.07%, *SD* = 1.52%, Z = 5.11, *p* < 0.001, Wilcoxon’s *r* = 0.88) as well as in the *mismatch* condition (Experiment 1: *M* = 83.86%, *SD* = 11.18%, *Z* = 5.04, *p* < 0.001, Wilcoxon’s *r* = 0.87; Experiment 2: *M* = 87.93%, *SD* = 10.65%, *Z* = 5.08, *p* < 0.001, Wilcoxon’s *r* = 0.87). In Experiment 1, the accuracies of both conditions did not differ significantly from each other (Experiment 1: *Z* = 1.41, *p* = 0.16, Wilcoxon’s *r* = 0.24), while, in Experiment 2, the accuracies in the *match* condition were higher compared to the accuracies in the *mismatch* condition (*Z* = 5.01, *p* < 0.001, Wilcoxon’s *r* = 0.86).

### Predictive saccades toward expected facial feature

Experiment 1 investigated whether participants use expectations to perform anticipatory eye movements. Indeed, expectations led to predictive saccades during the inter-stimulus interval (ISI) toward face locations associated with the expected distinct facial feature. A two-way ANOVA revealed the main effect of “expectation” (*F*(1,33) = 157.86, *p* < 0.001, *η*_*p*_^*2*^ = 0.83), as well as a significant interaction (*F*(1,33) = 14.27, *p* < 0.001, *η*_*p*_^*2*^ = 0.30). Specifically, participants performed more saccades to a region of interest (ROI) if the corresponding facial feature was expected (main effect “expectation”: expected: *M* = 51.70%, *SD* = 18.20%; unexpected: *M* = 7.22%, *SD* = 4.17%, Figures 3A and 3B). There was no main effect of “saccade” (*F*(1,33) = 0.01, *p* = 0.91, *η*_*p*_^*2*^ = 0.0004). We report the corresponding *post hoc t* tests (Bonferroni-corrected) for completeness. The first and second saccade landed more often in an ROI in trials in which its facial feature had been expected compared to when it had not been expected, respectively (first/expected: *M* = 50.30%, *SD* = 16.80%; first/unexpected: *M* = 8.71%, SD = 4.20%; *t*(33) = 11.98, *p* < 0.001, 95% CI [31.86, 51.35], *d* = 2.06; second/expected: *M* = 53.10%, *SD* = 19.70%; second/unexpected: *M* = 5.74%, *SD* = 3.63%; *t*(33) = 12.59, *p* < 0.001, 95% CI [36.77, 57.87], *d* = 2.16). Furthermore, this expectation effect was also evident when directly comparing the first and second saccade; i.e., the first saccade landed more often in an ROI if its facial feature had been expected compared to the second saccade in trials in which it had not been expected (first/expected vs. second/unexpected: *t*(33) = 13.24, *p* < 0.001, 95% CI [35.12, 54.03], *d* = 2.12). This was also evident for the reverse effect; i.e., the second saccade landed more often in an ROI in trials in which its facial feature had been expected compared to the first saccade in trials in which its facial feature had not been expected (second/expected vs. first/unexpected: *t*(33) = 11.23, *p* < 0.001, 95% CI [33.27, 55.43], *d* = 1.93). Lastly, for expected facial features, there was no difference in how often the first and second saccade landed in the respective ROI (first/expected vs. second/expected: *t*(33) = −1.63, *p* = 0.68, 95% CI [−7.47, 1.99], *d* = −0.28), while second saccades landed more frequently in the unexpected ROIs compared to first saccades (*t*(33) = 6.79, *p* < 0.001, 95% CI [1.74, 4.20], *d* = 1.16).

### Expectations guide fixations during face recognition

**Figure 4:**
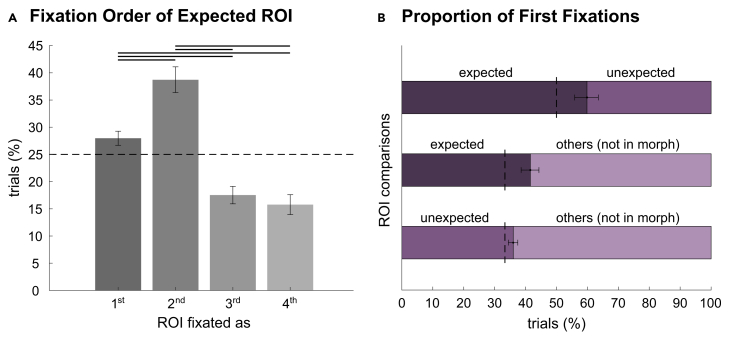
Results of Experiment 2 (A) Early fixation of the expected region of interest (ROI): in all trials (i.e., *match*, *mismatch*, *partial*), participants fixated the expected ROI more often as first or second out of all four ROIs. Error bars indicate 95% CIs. The dashed line represents the chance level (*π* = 0.25). Black lines indicate *p* < 0.001. (B) Fixation order in partial trials: in face morphs, participants fixated the ROI containing the expected feature earlier than the ROI containing the feature of the unexpected identity and the other two ROIs. They also fixated on the unexpected feature earlier than on the other two ROIs. Error bars indicate 95% CIs. The dashed lines represent the chance level (*π* = 0.50 and *π* = 0.33, respectively). See also Figure S3.

In Experiment 2, across all conditions (i.e., *match*, *mismatch*, and *partial*), expectations influenced the order in which the expected ROI was fixated (*χ*^*2*^(3, *N* = 34) = 27.47, *p* < 0.001, *V* = 0.03; Figure 4A). *Post hoc* tests revealed that this effect was mainly driven by the expected ROI being fixated first or second more often than third or fourth (first vs. third: *z* = 4.83, *p* < 0.001, 95% CI [0.57, 0.67], *h* = 0.24; first vs. fourth: *z* = 4.84, *p* < 0.001, 95% CI [0.59, 0.70], *h* = 0.31; second vs. third: *z* = 5.27, *p* < 0.001, 95% CI [0.62, 0.76], *h* = 0.39; second vs. fourth: *z* = 5.25, *p* < 0.001, 95% CI [0.63, 0.79], *h* = 0.45). The number of times the expected ROI was fixated first or second differed (*z* = −5.06, *p* < 0.001, 95% CI [0.39, 0.45], *h* = 0.16), whereas the number of times it was fixated third or fourth did not differ (*z* = 2.18, *p* = 0.17, 95% CI [0.50, 0.57], *h* = 0.15).

Similarly, in face morphs, the expected ROI was fixated earlier than the unexpected ROI (*z* = 4.98, *p* < 0.001, 95% CI [0.56, 0.64], *h* = 0.20) as well as the other ROIs containing no distinct feature (*z* = 5.54, *p* < 0.001, 95% CI [0.39, 0.44], *h* = 0.17). The unexpected ROI was also fixated earlier than the other ROIs (*z* = 3.37, *p* < 0.001, 95% CI [0.34, 0.37], *h* = 0.08, Figure 4B), indicating that, after initial guidance by expectations, bottom-up deviations influenced subsequent fixations.

In *mismatch* trials, the expectation guidance of initial fixations was partially counterbalanced by bottom-up information: While the expected ROI was still fixated earlier than the other two ROIs (*z* = 4.37, *p* < 0.001, 95% CI [0.36, 0.41], *h* = 0.11), it was not fixated earlier than the unexpected ROI (*z* = −1.71, *p* = 0.09, 95% CI [0.44, 0.50], *h* = 0.14). Rather, the unexpected ROI was also fixated earlier than the other two ROIs (*z* = 5.33, *p* < 0.001, 95% CI [0.37, 0.41], *h* = 0.12), suggesting that, if the sensory information distinctly differed from the expectation, when not a face morph but a completely unexpected face was presented, top-down as well as bottom-up information guided eye movements to informative locations.

The second aim of Experiment 2 was to investigate whether expectations influence how often and how long information is sampled from the expected and unexpected facial features. There were differences in the number of fixations and dwell time in line with a congruency effect; i.e., expected facial features in face morphs were fixated more often and longer than unexpected and other features (number of fixations: expected: *M* = 26.58%, *SD* = 3.91%; unexpected: *M* = 21.99%, *SD* = 2.78%; others: *M* = 15.34%, *SD* = 2.09%; expected vs. unexpected: *V* = 582, *p* < 0.001, 95% CI [2.82, 5.74], Wilcoxon’s *r* = 0.83; expected vs. others: *V* = 595, *p* < 0.001, 95% CI [9.53, 12.54], Wilcoxon’s *r* = 0.87; dwell time: expected: *M* = 22.86%, *SD* = 3.73%; unexpected: *M* = 18.84%, *SD* = 2.23%; others: *M* = 12.04%, *SD* = 1.91%; expected vs. unexpected: *V* = 559, *p* < 0.001, 95% CI [2.29, 5.38], Wilcoxon’s *r* = 0.77; expected vs. others: *V* = 595, *p* < 0.001, 95% CI [9.19, 12.20], Wilcoxon’s *r* = 0.87).

Further exploratory analyses of fixation time courses during face presentation revealed an interaction effect of expectation and time on the number of fixations and dwell times, with an initial preferred sampling of the expected feature in the first 1,000 ms of face presentation, which reversed to an increased sampling of the unexpected feature in the later time window of 1,500–2,000 ms (Figure S1). Participants differentially fixated the expected, unexpected, and other ROIs (main effect “ROI”: number of fixations: *F*(1.92, 63.20) = 138.94, *p* < 0.001, *η*_*p*_^*2*^ = 0.81; dwell time: *F*(1.88, 62.13) = 132.56, *p* < 0.001, *η*_*p*_^*2*^ = 0.80). In addition, there was a difference in the number of fixations toward any of the four ROIs over time (main effect “bin”: *F*(1.70, 55.98) = 9.80, *p* < 0.001, *η*_*p*_^*2*^ = 0.23), although this was not evident in dwell times (*F*(1.85, 60.99) = 1.94, *p* = 0.16, *η*_*p*_^*2*^ = 0.06). For both, i.e., number of fixations and dwell time, there was an interaction between “ROI” and “bin” (number of fixations: *F*(3.49, 115.09) = 26.07, *p* < 0.001 = 0.18, *η*_*p*_^*2*^ = 0.44; dwell time: *F*(3.53, 116.35) = 23.26, *p* < 0.001, *η*_*p*_^*2*^ = 0.41), showing a preferred sampling of the expected feature in the first two bins, which reversed into an increased sampling of the unexpected feature in the fourth bin (number of fixations: expected vs. unexpected: first bin: *t*(33) = 7.94, *p* < 0.001, 95% CI [5.97, 15.02], *d* = 1.36; second bin: *t*(33) = 5.47, *p* < 0.001, 95% CI [3.26, 14.20], *d* = 0.94; fourth bin: *t*(33) = −4.00, *p* = 0.01, 95% CI [−10.28, −0.80], *d* = −0.69; dwell time: expected vs. unexpected: first bin: *t*(33) = 6.77, *p* < 0.001, 95% CI [3.89, 11.85], *d* = 1.16; second bin: *t*(33) = 4.11, *p* = 0.008, 95% CI [1.09, 12.03], *d* = 0.70; fourth bin: *t*(33) = −4.08, *p* = 0.008, 95% CI [−9.04, −0.79], *d* = −0.70).

Moreover, an exploratory analysis of fixation durations showed that fixations on the expected and unexpected ROIs were longer compared to the other ROIs, reflecting an increased sampling of informative features in face morphs (Figure S2). There were differences in the fixation duration depending on expectation (main effect “ROI”: *F*(2, 66) = 44.63, *p* < 0.001, *η*^*2*^ = 0.122), with longer fixations on features distinctive for the two identities within a face morph compared to the other ROIs (expected: *M* = 239.58 ms, *SD* = 34.21 ms; unexpected: *M* = 244.88 ms, *SD* = 37.78 ms; others: *M* = 215.68 ms, *SD* = 31.67 ms; expected vs. others: *t*(33) = 7.15, *p* < 0.001, 95% CI [15.50, 32.32], *d* = 1.23; unexpected vs. others: *t*(33) = 9.09, *p* < 0.001, 95% CI [21.10, 37.30], *d* = 1.56). There was no difference in the fixation duration between the expected and unexpected ROI (*t*(33) = −1.59, *p* = 0.36, 95% CI [−13.70, 3.09], *d* = −0.27).

### Linking eye movements to behavior

Finally, we investigated the relationship between eye movements and participants’ responses. In Experiment 1, the accuracy in identifying a clear face as expected or unexpected was higher if participants fixated on the expected facial feature at face onset, in the *match* (*M* = 91.40%, *SD* = 9.22% vs. *M* = 76.40%, *SD* = 12.40%; *V* = 584, *p* < 0.001, 95% CI [10.82, Inf], Wilcoxon’s *r* = 0.84) and in the *mismatch* condition (*M* = 87.40%, *SD* = 11.90% vs. *M* = 76.80%, *SD* = 14.90%; *V* = 529, *p* < 0.001, [5.90, Inf], Wilcoxon’s *r* = 0.68). Interestingly, in *partial* trials containing face morphs, participants were more likely than chance level to indicate the expected face identity if they fixated on the expected ROI at face onset (*M* = 89.73%, *SD* = 14.25%; *V* = 592, *p* < 0.001, [87.31, Inf], Wilcoxon’s *r* = 0.86), possibly linking the assimilation effect to the gathering of expectation-compliant information at the expected face location.

In Experiment 2, there was a last-sampling bias; i.e., participants perceived the identity in a face morph more often than chance if they fixated its distinct ROI last (*z* = 7.09, *p* < 0.001, 95% CI [0.36, 0.45], *h* = 0.35).

## Discussion

In two preregistered eye-tracking studies, we investigated the influence of expectations, induced by name cues, on gazing behavior when viewing faces. We created ambiguous sensory information by morphing two faces containing expected as well as unexpected facial features. In the first experiment, participants performed predictive saccades toward expected facial features. In the second experiment, expectations guided fixations toward expected facial features in face morphs, which were reversed toward unexpected features over time. In both experiments, participants were faster in recognizing an expected presented face compared to unexpected or morphed ones. Furthermore, the name prior shifted the identification of the ambiguous face morphs toward the expected identity. Overall, our results show that context shapes information sampling during face recognition, particularly during the early examination of a face. Guiding the eyes toward locations with expected facial features may help to evaluate whether the provided sensory information matches the prediction. We thereby support the established view that perception is an active process in which bottom-up sensory information and top-down expectations are combined.^7^^,^^8^^,^^9^

In our first experiment, as hypothesized, participants performed predictive saccades toward expected facial features, effectively translating context information into anticipatory eye movements. While previous literature on eye movements during face perception showed that humans tend to initially fixate on a point slightly below the eyes^23^^,^^24^^,^^36^^,^^37^ and preferentially fixate on the eyes and mouth,^27^^,^^28^ our findings show that higher-order contextual effects can revoke this automatism during anticipation. Expectations guide fixations toward facial features that are more informative for face identification, as has been shown for familiar faces compared to unfamiliar ones.^33^ An important determinant of whether top-down information can predictively guide eye movements could be task relevance.^38^^,^^39^ In our experiment, predictive saccades toward expected features allowed the sampling of relevant information to perform the identification task, further highlighting the possibility of task-relevant expectations to modify typical viewing behavior of faces. Our findings of expectation-dependent eye movements have wider implications for our understanding of face perception within the framework of “predictive perception” and active inference.^10^ According to this view of the human brain as a pro-active Bayesian hypothesis tester, gaze control provides a strategy to actively sample evidence at locations where important and relevant information is anticipated.^40^^,^^41^ Our findings support the theoretical assumption that action, here in the form of saccadic eye movements, tests perceptual hypotheses.^10^ This active visual sampling may partly explain why the perception and early neural processing of face identity and facial expression are context dependent.^1^ Translating this effect to face recognition in everyday situations, this confirmatory gaze strategy would lead to faster recognition of expected faces, while potentially overlooking additional deviating information within a face, as indicated by the assimilation effect for face morphs in our study—unless deviating sensory information leads to a rejection of the perceptual hypothesis as in the current *mismatch* trials.

In our second experiment, we found an order effect of expectation; i.e., expected facial features were fixated early in all trials, as well as earlier than the unexpected facial feature in *partial* trials. This is in line with the results of our first experiment, showing an early guidance of eye movements by expectations. The first fixations on a face are especially important to determine how the face is identified.^23^^,^^42^ Similarly, there were reduced latencies to the first target fixation when an object’s semantic surroundings have been primed^43^ and when an object semantically fitted to its surroundings (e.g., a pot compared to a printer in a kitchen^14^), supporting the idea of an active sampling of expected information.^10^ However, there have also been several contradictory reports of an earlier fixation on semantically incongruent information.^21^^,^^44^^,^^45^^,^^46^ Even when controlling for low-level stimulus properties, scene-incongruent information was recognized faster and more accurately, which has been taken as evidence for the preferred processing of unexpected information.^47^ In *partial* trials, we observed earlier fixations of expected compared to unexpected features as well as of unexpected compared to the other two features. Correspondingly in *mismatch* trials, participants fixated on the unexpected facial feature earlier than on the other two ROIs, with no difference between the fixation order of the expected and the unexpected ROI. This implies that, if sensory information sufficiently differs from expectations, it can also attract eye movements to unexpected locations. We speculate that the weighted interplay of both bottom-up deviations and top-down context-driven goals may provide a useful strategy during visual sampling while less important deviations in the environment are investigated after processing of the expected information.

In line with the expectation effect on fixation order, we found a congruency effect on fixation number and dwell time; i.e., participants fixated on the expected facial feature more often and longer than on the unexpected facial feature in a face morph. An active sampling of expected information is in line with previous literature reporting more fixations on semantically congruent scenes^20^ but contradicts other experiments demonstrating longer dwell times on scene-incongruent information.^21^^,^^22^ Further exploratory analyses of fixation time courses in 500 ms bins revealed that this preference for the expected facial feature was especially evident during the first 1,000 ms, followed by increased fixations of the unexpected features during 1,500–2,000 ms.

On the behavioral level, we found the typical facilitation effect with faster RTs for expected compared to unexpected clear or morphed faces.^4^^,^^5^^,^^6^^,^^7^ Furthermore, there was an assimilation effect; i.e., participants indicated the expected identity more often in a face morph, as anticipated for the short cue duration,^7^ rather than contrastive after-effects in adaptation designs (for review, see Mueller et al.^48^).

Participants showed higher accuracy for identifying a presented face if they fixated on the expected feature at face onset (Experiment 1) and a last-sampling bias^49^; i.e., they chose an identity more often than chance if they fixated on its distinct ROI last (Experiment 2). These links between fixations and behavioral responses suggest that the perceptual decision about the identity of a face morph was reached by an information sampling strategy, in which first the expected informative features were tested and sampling at other locations continued until enough confirmatory information for the decision was obtained.^10^

Future research is needed to evaluate the ecological validity of expectations guiding viewing behavior in face identification. This could be realized in face-to-face experimental settings and mobile tracking of eye movements.^50^^,^^51^ Furthermore, viewing patterns of both super-recognizers, i.e., individuals who are exceptionally skilled in face identification,^52^^,^^53^ as well as developmental and acquired prosopagnosia, i.e., individuals who have difficulty recognizing familiar faces,^54^^,^^55^ bear the potential to gain more insights into human face recognition. Superior performance might be related to more efficient processing of informative facial features to identify faces, whereas patients with prosopagnosia may fixate on less informative facial regions.^54^^,^^56^^,^^57^ Our findings may also have broader implications for understanding atypical face-viewing behavior, such as that observed in individuals with autism spectrum disorders.^58^^,^^59^^,^^60^ Understanding the effect of different viewing strategies might provide the potential to train and improve face recognition skills by focusing on expected informative facial features and help to understand why observers deploy idiosyncratic strategies.^61^^,^^62^

Overall, we were able to show that context-induced expectations guide predictive saccades toward and an early sampling of expected features during face recognition. Hence, expectations can influence the way we look at expected faces and direct the extraction of visual information from them. In face morphs, after sampling of expected information, unexpected features captured eye movements, suggesting that bottom-up information is additionally considered during the perception of visually ambiguous sensory information. Our results offer compelling evidence that expectations shape sampling of visual information, contributing empirical evidence to the influential theoretical framework of predictive processing.

### Limitations of the study

Finally, some limitations of our experimental design should be acknowledged. Firstly, in both experiments, the name cue was task relevant as participants had to indicate whether a face was expected or unexpected. It will be interesting to test whether our observed expectation-dependent gaze patterns are also present in implicit task settings. Secondly, in Experiment 2, participants might have responded “expected” more frequently to shorten the duration of the experiment, possibly contributing to the behavioral assimilation effect. However, this should not affect the initial guidance of fixations. Thirdly, the manipulated facial features differed in saliency and could have affected gazing behavior in a purely bottom-up fashion during the second experiment. While this holds for the *match* trials, it does not explain the initial guidance to expected features in *partial* trials in which faces contained salient information in two regions. In *mismatch* trials, we observed preferred initial fixation of the expected and unexpected ROIs compared to the two regions of no interest, hinting toward a combination of bottom-up- and top-down-driven viewing behavior. Fourthly, the order analysis showed that overall the expected ROI was more often fixated second than first. This was due to first fixations landing predominantly in the center of the face, in line with a central viewing tendency^24^^,^^63^ (Figure S3). Nevertheless, expectations clearly guided the second fixation to the other three ROIs in contrast to later (i.e., third and fourth) fixations (Figure S3). Lastly, sample sizes were preregistered and based on power analyses to reach a power of at least 0.80 to find effects of medium size at an alpha error probability of 5%. Studies powered to detect small effect sizes may reveal whether the currently observed group differences are robust. We did not perform analyses split up by gender because we investigated a general perceptual effect and our experimental samples were not powered for gender-based analyses.

## Resource availability

### Lead contact

Further information and requests for resources and reagents should be directed to and will be fulfilled by the lead contact, Helen Blank (h.blank@uke.de).

### Materials availability

The face images and auditory feedback stimuli generated in this study are available via the Open Science Framework (OSF) (Experiment 1: https://osf.io/7e38v/; Experiment 2: https://osf.io/tbdh6/).

### Data and code availability

• All data have been deposited at the OSF and are publicly available as of the date of publication. DOIs are listed in the key resources table.
• All original code has been deposited at the OSF and is publicly available as of the date of publication. DOIs are listed in the key resources table.
• No additional information is required to reanalyze the data reported in this paper.

## Acknowledgments

This project was funded by the Emmy Noether program of the Deutsche Forschungsgemeinschaft (German Research Foundation; grant no DFG BL 1736/1-1 to H.B.). We would like to thank Fabian Schneider, Carina Ufer, and Janika Becker for constructive discussions and their comments on the figures. We acknowledge financial support from the Open Access Publication Fund of UKE - Universitätsklinikum Hamburg-Eppendorf.

## Author contributions

A.G.: conceptualization, methodology, formal analysis, data curation, writing – original draft, visualization, and project administration. M.L.: conceptualization, methodology, validation, investigation, and writing – review and editing. M.G.: conceptualization, methodology, resources, and writing – review and editing. H.B.: conceptualization, methodology, writing – original draft, supervision, project administration, and funding acquisition.

## Declaration of interests

The authors declare no competing interests.

## STAR★Methods

### Key resources table

**Table:** 

REAGENT or RESOURCE	SOURCE	IDENTIFIER
**Deposited data**		

Experimental data (Experiment 1)	own	https://doi.org/10.17605/OSF.IO/7E38V
Stimuli (Experiment 1)	own	https://doi.org/10.17605/OSF.IO/7E38V
Material for experiments (Experiment 1)	own	https://doi.org/10.17605/OSF.IO/7E38V
Code for analyses (Experiment 1)	own	https://doi.org/10.17605/OSF.IO/7E38V
Code for experiments (Experiment 1)	own	https://doi.org/10.17605/OSF.IO/7E38V
Experimental data (Experiment 2)	own	https://doi.org/10.17605/OSF.IO/TBDH6
Stimuli (Experiment 2)	own	https://doi.org/10.17605/OSF.IO/TBDH6
Material for experiments (Experiment 2)	own	https://doi.org/10.17605/OSF.IO/TBDH6
Code for analyses (Experiment 2)	own	https://doi.org/10.17605/OSF.IO/TBDH6
Code for experiments (Experiment 2)	own	https://doi.org/10.17605/OSF.IO/TBDH6

**Software and algorithms**		

R (v4.2.0)	R Core Team	https://www.r-project.org
RStudio (v2022.02.2)	RStudio Team	https://posit.co
MATLAB (vR2020b)	Mathworks	https://de.mathworks.com
faceMaker	Schwind et al.^63^	http://facemaker.uvrg.org/
Audacity (v3.0.0)	Audacity Team	http://audacity.sourceforge.net/

### Experimental model and study participant details

#### Sample size

For Experiment 1, we report the results of 34 participants (17 females, self-reported gender) with a mean age of 26.5 years (*SD* = 4.38 years) (see quantification and statistical analysis). For Experiment 2, 34 participants (18 females) with a mean age of 25.09 years (*SD* = 4.91 years) were included. Participants had no history of neurological or psychiatric disorders. All experimental procedures were approved by the Ethics Committee of the Chamber of Physicians in Hamburg and participants provided written informed consent. We did not perform analyses split up by gender because we investigated a general perceptual effect and our experimental samples were not powered for gender-based analyses.

### Method details

#### Apparatus and stimuli

Stimuli were presented on a Samsung SyncMaster 204B display (41.0 × 31.0 cm; 20.1″) with a resolution of 1600 × 1200 pixels and a refresh rate of 75 Hz. Eye movements were recorded from the participant’s right eye using an EyeLink 1000 system at a sampling rate of 1000 Hz. The head location of the participant was fixed using a chin rest and forehead bar. Saccades were defined as periods in which the velocity exceeded 30°/sec or the acceleration 8000°/sec^2^, respectively. The saccadic motion threshold was set to 0.1°. A 13-point-calibration was performed at the start of each experimental block in both experiments. Validation was repeated until the result was at least “good” according to the guidelines of the manufacturer (i.e., worst point error <1.5°, average error <1.0°). In case of calibration issues (e.g., due to dense eyelashes), a validated 9- or 5-point-calibration was used.

We used grey-scale images of four male faces created with FaceMaker (http://facemaker.uvrg.org/)^64^ (Figure 1A). The four faces differed in one distinct feature (forehead, chin, ears, or nose) from an average ‘base face’. For the ‘base face’, the parameters ‘eyebrowsColor’, ‘hairColor’, ‘faceGender’, and ‘skinColor’ were set to 1. The parameters of interest for creating different identities were set to: ‘foreheadHeight’ (0.7), ‘jawChin’ (0.575), ‘jawLength’ (0.55), ‘earSize’ (0.7), and ‘noseWidth’ (0.625). For the first identity with a high forehead, ‘foreheadHeight’ was set to 0.9 while keeping the other parameters constant. For the second identity with a wide chin, ‘jawChin’ was set to 0.65 and ‘jawLength’ to 0.6. For the third identity with large ears, ‘earSize’ was set to 0.9. For the fourth identity with a large nose, ‘noseWidth’ was set to 0.75. Six pairwise face morphs (50/50%) between all faces were created by adjusting the parameters of interest to the mean values between the base face and the two respective identities.The size of the images was 1100 × 1100 pixels, with the face covering approximately 1064 × 736 pixels of the screen. Distance from the eyes to the screen was ∼560 mm, leading to a visual angle of 27.44 × 19.17° for the face stimuli.

For auditory feedback, we used three different tones to indicate ‘correct’, ‘incorrect’, or ‘too slow’ responses. All tones were generated with Audacity 3.0.0 (http://audacity.sourceforge.net/) with a duration of 200 ms and an amplitude of 0.7. The ‘correct’ tone was a sinus wave (880 Hz), and the ‘incorrect’ and ‘too slow’ tones were square waves (no aliasing) with 440 Hz and 220 Hz, respectively. The loudness of the tones was normalised.

#### Training sessions

In the training sessions of both experiments, no eye-tracking data were collected. The head location of the participant was fixed using a chin rest and forehead bar to ensure the same distance to the monitor and visual input as in the main experiment.

The first training was identical for both experiments (∼15 min). Participants learned to associate the four distinct faces with their respective names (Ari, Bob, Cid, Dan). The training was divided into three blocks. In each trial of the first block (32 trials), a face was shown for 4500 ms. The task was to identify the face by pressing one out of four buttons with the right hand (index, middle, ring, and pinky finger). Afterward, feedback was provided by presenting the face with a red circle around its distinct feature, a tone indicating whether the response was correct, incorrect, or too slow, as well as written text at the bottom of the screen (‘correct’, ‘incorrect’, or ‘too slow’, and the correct name) for 3000 ms. Each face was shown eight times. In the second block (48 trials), the face presentation duration was shortened to 3500 ms, and visual as well as auditory feedback was provided for 2000 ms. Each face was shown 12 times. In the third block (48 trials), a face was presented for 3500 ms. Only auditory feedback was provided. The experimenter provided feedback about the accuracy score in the last block. If it was below 75%, the last training block was repeated up to two times. If the threshold could still not be surpassed, the participant did not proceed to the next training and the main experiment. In the second training, participants got accustomed to the task of the main experiment. For the training of Experiment 1, 12 trials were shown (∼1–2 min). Each name prior was shown three times, once followed by the expected face (match), an unexpected face (mismatch), and a face morph containing the expected identity (partial). For the training of Experiment 2, 24 trials were shown (∼2 min). Each prior name was shown six times, twice for each experimental condition (match, mismatch, partial). In between the experimental blocks (three and four blocks in Experiment 1 and 2, respectively), a short repetition of the first training was performed (∼2 min) to ensure that participants still had a clear mental representation of the four distinct faces (16 trials, each face four times).

#### Procedure

In Experiment 1, participants first learned to associate the four faces with their respective names in training. In each trial of the main experiment, a name prior (Ari, Bob, Cid, or Dan) was presented as a cue for the upcoming face in one of the four corners of the screen (750 ms) (Figure 1D). Face-name associations were counterbalanced across participants. In the inter-stimulus interval (ISI), a face outline of the ‘base face’ (white line) was shown (1000 ms) so that participants could anticipate where the face would appear, followed by a brief presentation of a face (100 ms) and a response window (1500 ms). The presented face was either one of the four learned identities or one of the six pairwise morph combinations (e.g., 50/50% morph between Bob and Cid, Figure 1B). The task was to indicate with one of two buttons whether the presented face was either ‘expected’ or ‘unexpected’ based on the prior name. The allocation of the response options to the left and right keys was counterbalanced across participants. Auditory feedback indicated whether the response was correct, incorrect, or too slow. For face morphs, any response was counted as correct. The experiment was divided into three blocks with 144 trials each (∼13 min per block). The ratio of trials per condition, i.e., *match* (face matched to the name), *mismatch* (face did not match to the name), and *partial* (face morph containing the expected and an unexpected face identity), was identical in all blocks (48 trials per condition). The trial order within each block was pseudo-randomized, ensuring that the same name cue was restricted to consecutively appear twice at maximum. Each name prior appeared equally often in the upper-left, upper-right, bottom-left, and bottom-right corner. The total duration of the experiment was approximately 90 min.

For Experiment 2, the face was presented longer than in Experiment 1 to allow for visual exploration. Participants started by learning the four identities and their distinct features. In the main experiment, as in Experiment 1, a name was randomly presented in one of the four corners of the screen, followed by the presentation of a clear or morphed face (Figure 1E). The face images were either presented for 4500 ms or until the button press. The first task in each trial was to indicate whether the presented face was ‘expected’ or ‘unexpected’ based on the preceding name. If participants answered ‘unexpected’, a question mark prompted them to respond which identity was perceived in the face. Auditory feedback for the first and second tasks was only provided for too-slow responses. Each trial lasted either maximally 7500 ms (in case of responding ‘expected’) or 9000 ms (in case of responding ‘unexpected’). The experiment was divided into four blocks (∼10 min). The ratio of trials per condition (*match*, *mismatch*, *partial*) was identical in all blocks (72 trials per block). In addition to the pseudo-randomization and counterbalancing described for Experiment 1, the question mark was randomly presented in one of the four corners of the screen. The total duration of the experiment was approximately 90 min.

### Quantification and statistical analysis

Analyses were performed using custom-written scripts in R/RStudio (https://www.r-project.org; https://posit.co) and MATLAB R2020b (https://de.mathworks.com). In case data were not normally distributed (Shapiro-Wilk tests, *p* < 0.05, and visual inspection of Q-Q plots) or outliers were present in the data (above or below Q_1/3_−/+1.5∗IQR), we calculated non-parametric tests instead of the preregistered parametric tests, except for the ANOVAs due to their robustness to slight violations of the normality assumption and a lack of non-parametric alternatives for two-factorial designs.

#### Data exclusion and sample size estimation

For Experiment 1, we measured 42 participants. Eight participants were excluded from final data analyses: two participants did not sufficiently learn the four identities and their features in the training (<75% accuracy) and six participants had less than 70% valid eye tracking trials, leading to a final sample of 34 participants. For Experiment 2, we measured 35 participants, one participant was excluded due to behavioral performance below chance level. Sample sizes for both experiments (*N* = 34, each) were preregistered and based on power analyses using G∗Power.^65^ For Experiment 1, the sample size was optimised to obtain 0.80 power to detect a medium effect size (*f* = 0.25) at 0.05 alpha error probability for testing whether participants perform more predictive saccades to a region of interest (ROI) if its facial feature has been expected compared to when it has not been expected, as the main effect ‘expectation’ in a 2 ✕ 2 repeated measures ANOVA with the within-subject factors ‘expectation’ (expected, unexpected) and ‘saccade’ (first, second). For Experiment 2, we aimed to optimise the power for our main research questions regarding the *partial* trials (face morphs): we tested whether the dwell time on the expected ROI was significantly different from the dwell time on (1) the ROI associated with the other identity contained in a face morph, and on (2) the other ROIs using paired *t*-tests (two-sided) with a power of 0.80 to detect a medium effect size (*dz* = 0.50) at 0.05 alpha error probability.

Data exclusion criteria were preregistered. Firstly, single trials were excluded if participants did not fixate the name prior because awareness of the prior is a prerequisite for the hypotheses about prediction-guided saccades and fixations we aimed to test. Secondly, trials with no response were excluded from data analyses. Thirdly, single trials were excluded if the loss of eye-tracking data, e.g., due to blinks, in our time windows of main interest exceeded a certain threshold. For Experiment 1 (‘predictive saccades’), trials were excluded if data loss during the ISI (face outline for 750 ms) exceeded 30%. In Experiment 2 (‘information sampling’), trials were excluded if less than 50% valid fixation data were available during face presentation. Face presentation duration was variable in each trial and was determined by a button press of the participant or was 4500 ms at maximum.

Whole datasets (i.e., participants) were excluded in case of too many excluded trials (>30%).^66^^,^^67^ This was the case for six participants in Experiment 1. Whole datasets were also excluded if the behavioral performance during the main experiment was below the chance level in the match and mismatch conditions. In Experiment 1, chance level accuracy was at 50% (correctly responding ‘expected’ or ‘unexpected’). One participant had an average accuracy score of 25% as well as > 30% invalid trials and was therefore excluded from final data analyses. In Experiment 2, the chance level for mismatch was at 12.5%: participants needed to first correctly indicate that the presented face was ‘unexpected’ and then correctly identify it was one out of four identities (0.5∗0.25 = 0.125). For match, we considered a response correct if participants either answered ‘expected’, or answered ‘unexpected’ but then correctly identified the face afterward. We chose the more conservative chance level of 0.5, also for the average match and mismatch scores. One participant in Experiment 2 had a mean accuracy score of 42.71% and was therefore excluded.

In addition to the preregistered exclusion criteria, a learning criterion during the training was important for the completion of the main experiment, making sure that participants learned the four identities and their respective distinct facial features. Participants needed at least 75% accuracy in identifying the four faces in the training to proceed to the main experiment. In Experiment 1, two participants were not able to surpass this cut-off despite two repetitions of the training phase (mean accuracies per participant: 22.92% and 65.97%) and did not participate in the main experiment.

#### Behavioral analyses

In both experiments, participants were asked to indicate whether a presented face was ‘expected’ or ‘unexpected’ given the preceding name. Firstly, a facilitation effect due to expectation, i.e., shorter reaction times (RT) for expected compared to unexpected clear and morphed faces, was tested with repeated-measures ANOVAs with the within-subject factor ‘condition’ (*match*, *mismatch*, *partial*). We report Greenhouse-Geisser corrected results due to sphericity violation as well as the generalised *η*^*2*^ as a measure of effect size. Pairwise comparisons were based on post-hoc tests (Bonferroni-corrected) with Cohen’s *d* as effect size estimate.

Secondly, we tested whether expectations shifted the perception of face morphs, either into the direction of the expected face (assimilation effect) or the unexpected face part (contrastive effect). For each participant, we calculated a perceived face identity score indicating which identity has been perceived in face morphs (*partial* condition) depending on the preceding name prior. For each face morph and name combination (e.g.,A_prior_AB_morph_), the number of expectation-noncompliant button responses was subtracted from the number of expectation-compliant responses and divided by the number of possible combinations (*N* = 12). This index was converted to percentage values (0–100%): A difference score of zero (no prior effect on morph identification) was converted to 50%, i.e., the participant equally often identified face morphs as the expected or unexpected identity. Values above 50% were indicative of an assimilation effect and values below 50% of a contrastive effect. On the group level, we tested against chance level using a one-sample *t*-test (two-sided).Thirdly, for both experiments, we calculated accuracy scores for the *match* and *mismatch* conditions. On the one hand, we tested each condition’s accuracy scores against chance level using one-sample Wilcoxon signed rank tests (one-sided): In Experiment 1, the chance level equaled a probability of 50%. In Experiment 2, the chance level for *mismatch* trials equaled a probability of 12.5% (first task: ‘unexpected’; second task: answering the correct ID out of four possibilities → 0.5∗0.25 = 0.125). For *match* trials, we considered trials as correct if participants either responded ‘expected’ or ‘unexpected’ and then correctly identified the person. We used the more conservative probability of 50% as chance level for the *match* condition. On the other hand, we tested whether there was a difference in accuracy scores between the two conditions using paired Wilcoxon signed rank tests (two-sided).

#### Eye-tracking analyses

The aim of Experiment 1 was to investigate whether participants use context to perform predictive eye movements if limited sensory information is available. We hypothesised to see predictive saccades during the ISI between name prior and face on the white face outline. We investigated the target locations of the first two saccades^68^ and introduced a minimum latency criterion of 100 ms after face outline onset to exclude non-intentional saccades. Our ROIs were four rectangular regions with the same area, covering the distinct features of the four identities (forehead, chin, ears, and nose; Figure 1C). We computed a 2 ✕ 2 repeated measures ANOVA with the within-subject factors ‘expectation’ (expected, unexpected) and ‘saccade’ (first, second). We tested whether significantly more first or second saccades were performed toward the ROI with the expected facial feature (hereafter: ‘expected ROI’) compared to how often the ROI was fixated in trials in which its facial feature was not expected. We calculated relative frequencies to account for the different number of trials in which the facial feature of an ROI (e.g., the nose) was expected vs. unexpected (1:3). Relative frequencies (%) of the different ROIs were averaged, yielding mean percentages for each participant for the different combinations of ‘saccade’ and ‘expectation’. Post-hoc tests (Bonferroni-corrected) were performed.

The aim of Experiment 2 was to investigate whether context influences the order of fixations as well as the time spent looking at expected or unexpected facial features. We used three measures to evaluate an expectation bias: (1) Order and ordinal number of fixations, (2) number of fixations, and (3) dwell time.^69^^,^^70^^,^^71^ To evaluate an order effect (1), we conducted two analyses: firstly, in all trials (i.e., *match*, *mismatch*, *partial*), we tested whether participants fixated the expected ROI earliest out of all the four ROIs. We considered trials in which at least one of the ROIs was fixated and assigned ordinal numbers of 1–4. In case of missing fixations, ROIs that were not fixated were randomly assigned to one of the missing ordinal numbers in that trial.^70^ We evaluated whether the distribution of ordinal numbers differed from a uniform distribution using a subject-level chi-square goodness of fit test (R-package {htestClust}^72^) and calculated Cramer’s *V* as an effect size. Post-hoc proportional tests for clustered data were performed, Bonferroni-corrected for the number of tests (*N* = 6). As an estimate of effect size, we averaged the subject-level Cohen’s *h*. Secondly, in *partial* and *mismatch* trials, separately, we tested whether the expected ROI, the ROI with the unexpected facial feature (hereafter: ‘unexpected ROI’), or the other two ROIs (hereafter: ‘other ROIs’) were fixated more often before the other by calculating tests of marginal proportion for clustered data against binomial distributions,^72^ tested against *π* = 0.5 and *π* = 0.33, respectively, to account for the twice as large area of the other ROIs. We considered trials in which at least one of the respective ROIs had been fixated. Next, we evaluated an expectation effect on the number and duration of fixations (2 and 3): We conducted paired Wilcoxon signed-rank tests (two-tailed) to test whether the number of fixations and/or the dwell time on the expected ROI differed compared to the unexpected ROI and the average across the other ROIs. The number of fixations and dwell times on each ROI were divided by the total number of fixations and face presentation duration, respectively, to yield proportions.

#### Number of fixations and dwell time analysis by bin

We explored whether expectations influenced information sampling in face morphs in early time windows of the presented face (Figure S1). Therefore, we analyzed the relative number of fixations and dwell time on the expected, unexpected, and other ROIs by splitting the face presentation duration into time windows of 500 ms. As the presentation duration in each trial depended on the response given by participants, data availability for different time windows varied. We considered the first four time windows (i.e., 2000 ms) given that the fourth window was the latest window in which every participant had more than one trial for data analysis. We conducted 3 ✕ 4 repeated-measures ANOVAs with the within-subject factors ‘ROI’ (expected, unexpected, others) and ‘bin’ (500 ms steps) on the number of fixations and dwell times as the dependent variables. We report Greenhouse-Geisser corrected results as well as partial η^2^. Post-hoc tests were Bonferroni-corrected and restricted to comparisons of interest, i.e., across conditions within each time window and within conditions across time windows.

#### Fixation durations

We explored fixation durations and conducted a one-way repeated-measures ANOVA with the within-subject factor ‘ROI’ (expected, unexpected, others) on average fixation durations as the dependent variable. We report *η*^*2*^ as a measure of effect size. Post-hoc tests were Bonferroni-corrected.

#### Combined behavioral and eye-tracking analyses

Lastly, we investigated whether there was a link between eye movements and the responses given by the participants. In Experiment 1, we tested whether accuracy for identifying faces as expected or unexpected (in *match* and *mismatch* trials) was higher in trials in which participants fixated the expected ROI at face onset compared to when they did not fixate it in paired Wilcoxon signed-rank tests (one-sided). Correspondingly, we investigated whether participants chose the expected identity in a face morph if they fixated the expected vs. the unexpected ROI at face onset using a Wilcoxon signed-rank test (one-sided).

In Experiment 2, we tested whether participants more often chose the identity in a face morph if they fixated their distinct ROI last^49^ using a proportional test for clustered data against chance level (*π* = 0.25).^72^

### Additional resources

The hypotheses, methods, and analysis plan were preregistered via the OSF (Experiment 1: https://osf.io/c2ydh; Experiment 2: https://osf.io/vxyrg). For minor deviations from these preregistrations see the detailed description in Tables S1 and S2 (template by Willroth and Atherton^73^).
