# Supplementary material for: Expectations guide predictive eye movements and information sampling during face recognition
Source: iScience. 2024 Sep 10;27(10):110920. doi: 10.1016/j.isci.2024.110920 (PMC11439840; doi:10.1016/j.isci.2024.110920)
Supplement: Document S1. Figures S1–S3 and Tables S1 and S2 [file mmc1.pdf]

iScience, Volume *27*

Supplemental information

Expectations guide predictive eye movements and information sampling during face recognition

Annika Garlichs, Mark Lustig, Matthias Gamer, and Helen Blank

Supplemental Tables

Table S1. Preregistration Deviations for Experiment 1, related to STAR Methods.

Deviations

#	Details		Original Wording	Deviation Description	Reader Impact
1	Type	Sample	Inclusion criterion: “German as first language”	During participant recruitment, we considered this limitation as too strict and reformulated it to “fluent German skills” in the adverts. Furthermore, we included one participant who spoke English by translating the instructions.	By choosing less strict inclusion criteria, the results are more likely to represent a broader population. We repeated all analyses excluding the one English speaker, revealing that this did not influence the results.
	Reason	Other (Please Explain)			
	Timing	During data collection			
2	Type	Other (Please Explain)	“At the beginning of each block, a 9-point-calibration is performed.“	We used a 13-point-calibration, if possible. In case of calibration issues (e.g., due to dense eyelashes), we used a validated 9- or 5-point-calibration.	A calibration with fewer points might lead to decreased accuracy of measured eye movements. In the final sample, a 9-point-calibration was used for four participants, and a 5-point-calibration for one participant.
	Reason	New knowledge			
	Timing	During data collection			
3	Type	Analysis	Behavioural analyses: “Secondly, we will test whether there is a significant difference in the accuracy scores of the conditions match and mismatch using a paired t-test. Furthermore, we will test the accuracy scores of	We used a paired Wilcoxon signed rank test (two-sided) as well as one-sample Wilcoxon signed rank tests (one-sided) to evaluate significance. The difference scores of *match* and *mismatch* accuracies were not	The non-parametric tests might have led to a decrease in power, making it harder to find an effect.<br><br>A paired *t*-test (two-sided) would have led to the same non-significant
	Reason	New knowledge			
	Timing	After results known			

			both conditions against chance level (.50) using one-sample t-tests (one-sided)."	normally distributed (Shapiro-Wilk test: $p = .01$), and neither were the accuracies of the conditions themselves (*match*: $p = .0011$; *mismatch*: $p < .001$). Furthermore, there were outliers present (*match*: $Z = -3.23$, $Z = -1.86$, $Z = -1.77$, $Z = -1.68$, $Z = -1.48$; *mismatch*: Z = -3.96, Z = -2.52).	result: $t(33) = 1.24$, $p = 0.22$, 95% CI [-1.65, 6.79].<br><br>One-sample *t*-tests (one-sided) would have led to the same significant results: *match*: $t(33) = 21.58$, $p < .001$, [83.57, Inf]; *mismatch*: $t(33) = 17.66$, $p < .001$, [80.62, Inf].
4	Type	Other (Please Explain)	Eye-tracking analysis: "2 ╳ 2 repeated measures ANOVA with the within-subject factors saccade number (first, second) and priming (primed, not primed)"	We renamed the factor priming (primed, not primed) to expectation (expected, unexpected) for coherent wording in our manuscript.	This does not affect the results.
	Reason	Other (Please Explain)			
	Timing	After results known			
5	Type	Analysis	Combined behavioural and eye-tracking analysis: "We will investigate whether participants have significantly higher accuracies for match and mismatch trials in which they predictively fixated the ROI with the expected face property at face onset compared to trials in which they did not fixate it. For testing significance, we will use paired t-tests (one-sided)."	We used paired Wilcoxon signed rank tests (one-sided) due to outliers in the data (*match*: ROI fixated: $Z = -3.59$, $Z = -2.31$, $Z = -1.99$; *mismatch*: ROI fixated: $Z = -4.10$, $Z = -2.44$; ROI not fixated: $Z = -2.47$, $Z = -1.98$).	The non-parametric tests might have led to a decrease in power, making it harder to find an effect.<br><br>Paired *t*-tests (one-sided) would have led to the same significant results (*match*: $t(33) = -7.60$, $p < .001$, 95% CI [11.73, Inf]; *mismatch*: $t(33) = 4.62$, $p < .001$, [6.74, Inf]).
	Reason	New knowledge			
	Timing	After results known			

Unregistered Steps

#	Details	Original Wording	Unregistered Step Description	Reader Impact

1	Type	Sample	Not preregistered	Exclusion criterion: During data collection, we noticed that few participants did not sufficiently learn the four identities and their distinct features in the training. We defined a cut-off threshold of 75%. We allowed participants to repeat the last part of the training up to two times. Two participants were not able to proceed to the main experiment (accuracies of the second repetition: 25.00% and 68.75%).	The additional exclusion criterion might have enhanced the results by preventing participants who did not learn the identities from proceeding to the main experiment.
	Timing	During data collection			
2	Type	Analysis	Not preregistered	Combined behavioural and eye-tracking analysis: We exploratively investigated “whether participants chose the expected identity in a face morph if they fixated the expected vs. the unexpected ROI at face onset using a Wilcoxon signed rank test (one-sided).” This additional analysis provides valuable insights into the possible source of the observed assimilation effect.	The result provides an additional interpretation possibility for the assimilation effect.
	Timing	After results known			

Note. Preregistration Deviation Table Template provided by Willroth and Atherton (Willroth EC, Atherton OE. Best Laid Plans: A Guide to Reporting Preregistration Deviations. *Advances in Methods and Practices in Psychological Science*. 2024;7(1). doi:10.1177/25152459231213802)

Table S2. Preregistration Deviations for Experiment 2, related to STAR Methods.

Deviations

#	Details		Original Wording	Deviation Description	Reader Impact
1	Type	Sample	Inclusion criterion: “German as first language”	During participant recruitment, we considered this limitation as too strict and reformulated it to “fluent German skills” in the adverts.	By choosing less strict inclusion criteria, the results are more likely to represent a broader population.
	Reason	Other (Please Explain)			
	Timing	During data collection			
2	Type	Study Design	“At the beginning of each block, a 9-point-calibration is performed.“	We used a 13-point-calibration, if possible. In case of calibration issues (e.g., due to dense eyelashes), we used a validated 9- or 5-point-calibration.	A calibration with fewer points might lead to decreased accuracy of measured eye movements. In the final sample, a 9-point-calibration was used for two participants, a 5-point-calibration for one participant.
	Reason	New knowledge			
	Timing	During data collection			
3	Type	Analysis	“Secondly, we will test whether there is a significant difference in the accuracy scores of the conditions match and mismatch using a paired t-test. Furthermore, we will test the accuracy scores of both conditions against chance level (.50) using one-sample t-tests (one-sided).”	Behavioural analyses: We used a paired Wilcoxon signed rank test (two-sided) as well as one-sample Wilcoxon signed rank tests (one-sided) to evaluate significance. The difference scores of *match* and *mismatch* accuracies were not normally distributed (Shapiro-Wilk test: $p < .001$), neither were the accuracies of the conditions themselves (*match*: $p = .009$; *mismatch*: $p < .001$).	The non-parametric tests might have led to a decrease in power, making it harder to find an effect. A paired *t*-test (two-sided) would have led to the same significant result: $t(33) = 5.83$, $p < .001$, 95% CI [6.60, 13.68]. One-sample *t*-tests (one-sided) would have led to the same significant results: *match*: $t(33) = 184.40$, $p < .001$, [97.63, Inf]; *mismatch*: $t(33) = 20.76$, $p < .001$, [84.84, Inf].
	Reason	New knowledge			
	Timing	After results known			
4	Type	Analysis	“For partial match trials, we will test whether (a) participants spend a significantly different	Eye-tracking analyses: Instead of averaging over the three ROIs not containing the expected feature, we computed	This increased the clarity of our statistical analyses, ruling out confounds of bottom-up information in
	Reason	New knowledge			

	Timing	After results known	dwell time on the expected ROI compared to the average proportion of dwell time on all other three ROIs. We will further test whether (b) participants spend a significantly different dwell time on the expected ROI compared to the ROI with the face property of the other identity in the face morph." … "Secondly, we will compare the proportion of fixations on the ROI with the expected face property to the proportion of fixations (a) on the average of all other ROIs, and (b) on the ROI with the face property of the other identity in the face morph. We will use paired t-tests (two-tailed) to test for significance."	comparisons between the expected ROI and the unexpected ROI or the other two ROIs. respectively. The unexpected ROI is qualitatively different from the other two ROIs not containing any salient information. Furthermore, we used paired Wilcoxon signed rank tests (two-sided) to evaluate significance due to outliers in the data (number of fixations: expected: $Z = 2.97$; other two: $Z = 2.77$, $Z = 2.17$; dwell time: other two: $Z = 2.71$, $Z = 2.56$). For comparability, we also calculated a Wilcoxon test for dwell time 'expected vs. unexpected'.	an averaging over the other ROIs. Paired t-tests would have led to the same results:<br>Number of fixations: expected vs. unexpected: $t(33) = 6.73$, $p < .001$, 95% CI [3.20, 5.98], $d = 1.15$; expected vs. other two: $t(33) = 15.12$, $p < .001$, 95% CI [9.73, 12.75], $d = 2.59$. Dwell time: expected vs. unexpected: $t(33) = 5.96$, $p < .001$, 95% CI [2.65, 5.40], $d = 1.02$; expected vs. other two: $t(33) = 14.75$, $p < .001$, 95% CI [9.33, 12.32], $d = 2.53$.

Unregistered Steps

1	Type	Sample	Not preregistered	Exclusion criterion: During data collection of the first experiment, we noticed that some participants did not sufficiently learn the four identities and their distinct features in the training. We defined a cut-off threshold of 75%. We allowed participants to repeat the last part of the training up to two times. All participants proceeded to the main experiment.	This did not affect the results as no participant was excluded.
	Timing	During data collection			
2	Type	Analysis	Not preregistered		

	Timing	After results known		Eye-tracking analysis: We investigated in *mismatch* trials whether the expected or unexpected ROI were fixated more often before each other and more often before the other two ROIs, respectively. We realised that the mismatch trials allowed us to disentangle whether expectations would guide eye movements even in the presence of unexpected salient features.	The *mismatch* trials provide valuable insights into whether bottom-up saliency can counterbalance top-down expectations in guiding participants' eye movements.
3	Type	Analysis	Not preregistered	Eye-tracking analysis: We investigated whether fixation order in each of the four ROIs differed depending on expectation.	This analysis allowed us to show that the first fixations predominantly landed in the nose ROI, followed by expectations guiding the second fixation.
	Timing	After results known			
3	Type	Analysis	Not preregistered, suggested during revision	Eye-tracking analysis: We explored early viewing patterns towards the expected, unexpected, and other two ROIs in the first 2000 ms, split up into bins of 500 ms. We computed two 3 $\times$ 4 repeated-measures ANOVAs with the within-subject factors 'ROI' (expected, unexpected, others) and 'bin' (500 ms steps), on the number of fixations and dwell times as the dependent variables.	This analysis allowed us to investigate whether the congruency effect in the number of fixations and dwell times, i.e., a preferred sampling of the expected facial feature in a face morph, varied within the face presentation duration.
	Timing	Other (Please Explain)			
4	Type	Analysis	Not preregistered, suggested during revision	Eye-tracking analysis: We explored whether single fixation durations towards the expected, unexpected, and other two ROIs were influenced by expectation. We conducted a one-way repeated-measures ANOVA with the within-subject factor 'ROI' (expected, unexpected, others) on average fixation durations (ms) as the dependent variable.	This analysis allowed us to investigate whether the processing of unexpected compared to expected facial features is linked to higher difficulty and cognitive processing.
	Timing	Other (Please Explain)			

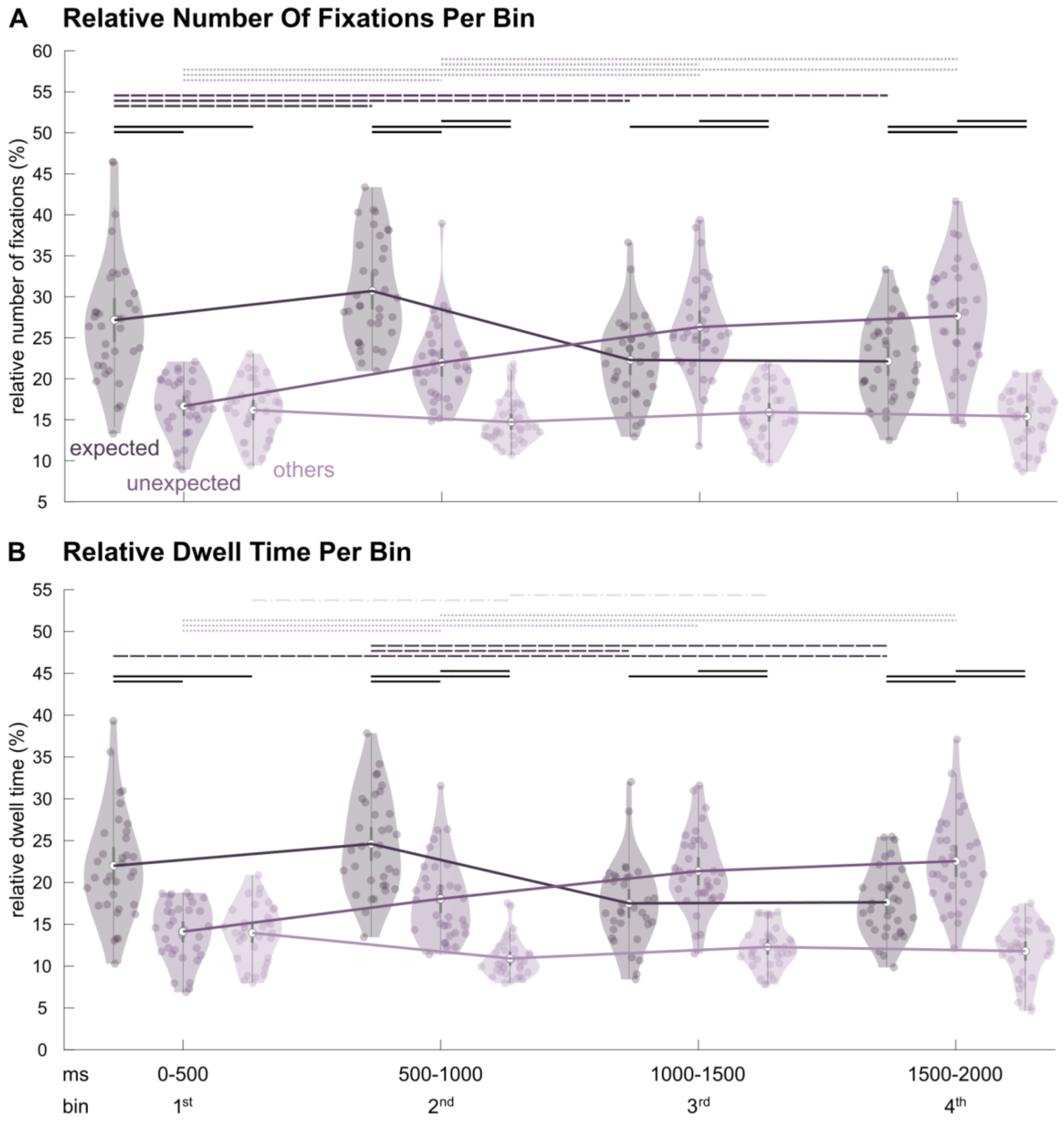

Figure S1. Early viewing of face morphs in Experiment 2, related to STAR Methods. A) Relative number of fixations: Effects of expectation on early viewing behaviour were investigated by splitting the first 2000 ms of face presentation in *partial* trials into bins of 500 ms. There was an interaction between 'ROI' and 'bin', showing that after initial preferred sampling of the expected region of interest (ROI) in the first and second bin, participants preferentially fixated on the unexpected ROI in the fourth bin. Dots represent single participants, white dots means, grey rectangles 95% confidence intervals (CI), and the lower and upper whiskers $Q_{1/3}$ -/+ 1.5*IQR. Post-hoc tests were Bonferroni-corrected for the comparisons of interest, i.e., across conditions within each time window and within conditions across time windows Lines indicate $p < .05$ (black/solid: across conditions, within bin; dark purple/dashed: within 'expected', across bins; rose/dotted: within 'unexpected', across bins. **B) Relative dwell time**: Similar to the relative number of fixations, dwell time analyses revealed an interaction between 'ROI' and 'bin'. Light rose/dashed-dotted: within 'others', across bins.

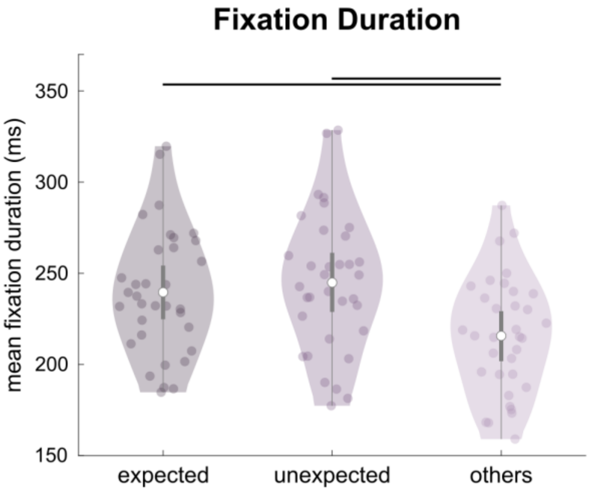

Figure S2. Average fixation duration for face morphs in Experiment 2, related to STAR Methods. For each participant, average fixation durations (ms) for the expected, unexpected, and other two ('others') regions of interest (ROI) were calculated. Dots represent single participants, white dots means, grey rectangles 95% confidence intervals (CI), and the lower and upper whiskers $Q_{1/3}$ -/+ 1.5*IQR. Black lines indicate $p < .05$.

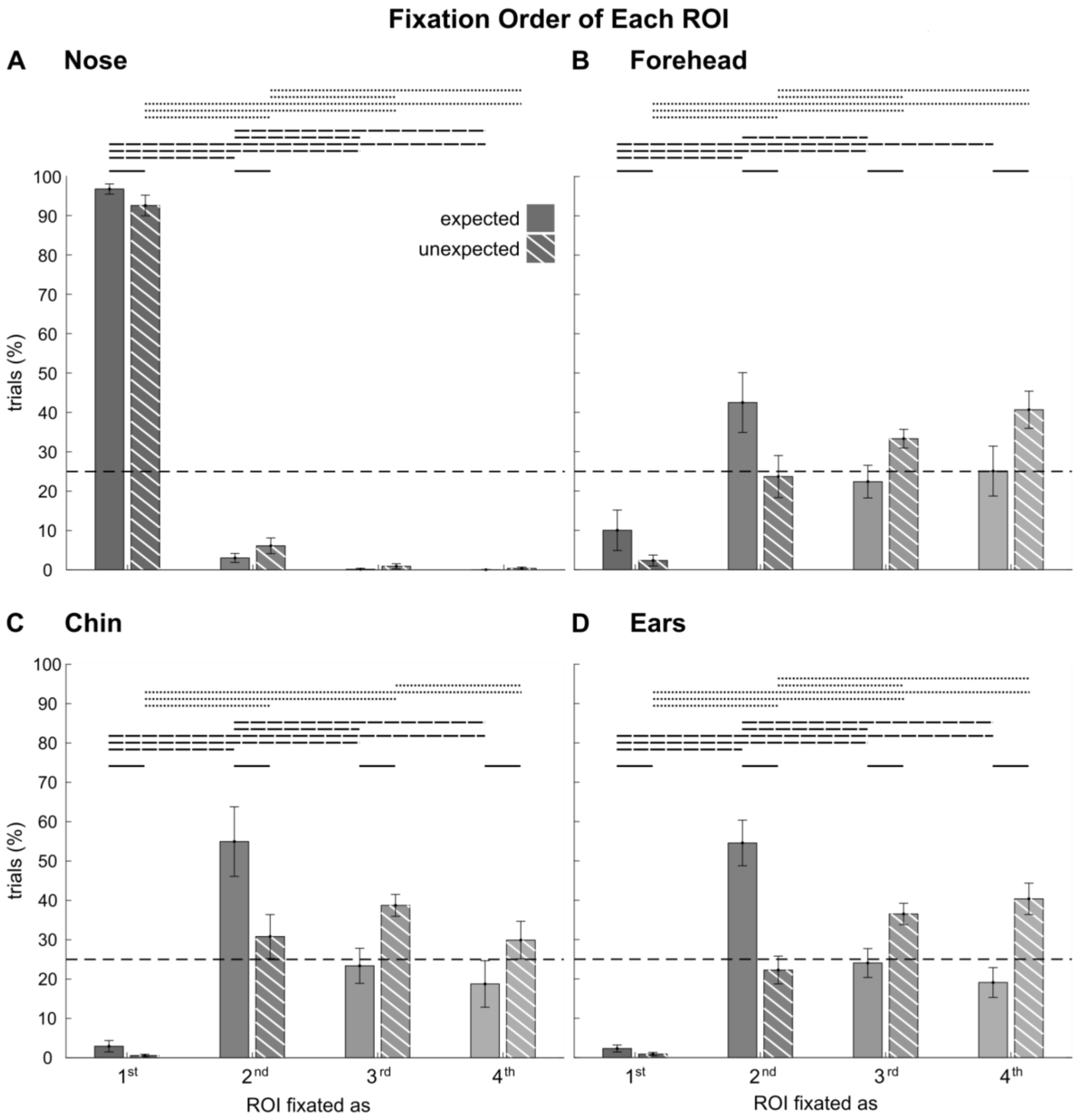

Figure S3. Order of fixations on the four regions of interest (ROI), related to Figure 4A. Depicted are percentages of how often each ROI was fixated as the first, second, third, or fourth out of all ROIs (**nose (A)**, **forehead (B)**, **chin (C)**, and **ears (D)**). Full and striped bars represent trials in which the ROI was expected or not expected, respectively. First fixations landed predominantly in the nose ROI, in line with a central viewing tendency. In the other three ROIs, expectations guided the second fixation towards an ROI. Error bars indicate 95% CIs. The dashed line represents the chance level ($\pi = .25$). Lines at the top indicate $p < .05$ (solid: expected vs. unexpected within each order number; dashed: within the condition ‘expected’; dotted: within the condition ‘unexpected’). The order analysis revealed that expectations guided eye movements towards the expected ROI so that it was fixated first or second more often than third or fourth (Figure 4A). In the following exploratory analysis, we further investigated whether fixation order differed depending on the ROI. In case of missing fixations within a trial, random ordinal numbers were assigned as described in the STAR Methods. Using subject-level chi-square goodness of fit tests, we evaluated whether the distribution of ordinal numbers differed from a uniform distribution within each ROI, for trials in which the ROI was either expected or unexpected. Cramer’s *V* was calculated as an effect size. Post-hoc proportional tests for clustered data were performed within

each ROI, Bonferroni-corrected for the number of tests (expected: $N = 6$; unexpected: $N = 6$; expected vs. unexpected: $N = 4$). In case of missing values in both conditions of interest, post-hoc tests were performed with the remaining participants. As an estimate of effect size, we averaged the subject-level Cohen's *h*. The order in which a ROI was fixated differed, both when its facial feature was expected (nose: $\chi^2(3, N = 34) = 34.00$, $p < .001$, $V = 0.07$; forehead: $\chi^2(3, N = 34) = 23.37$, $p < .001$, $V = 0.06$; chin: $\chi^2(3, N = 34) = 33.21$, $p < .001$, $V = 0.07$; ears: $\chi^2(3, N = 34) = 33.61$, $p < .001$, $V = 0.07$), and when its feature was unexpected (nose: $\chi^2(3, N = 34) = 33.97$, $p < .001$, $V = 0.04$; forehead: $\chi^2(3, N = 34) = 33.09$, $p < .001$, $V = 0.04$; chin: $\chi^2(3, N = 34) = 33.98$, $p < .001$, $V = 0.04$; ears: $\chi^2(3, N = 34) = 33.93$, $p < .001$, $V = 0.04$). First fixations landed predominantly in the nose ROI, especially if it was expected ($z = 3.51$, $p = .002$, 95% CI [0.25, 0.26], $h = 1.03$). After this initial fixation, participants fixated second on the expected ROIs (expected vs. unexpected: 2nd: forehead: $z = 6.42$, $p < .001$, 95% CI [0.35, 0.44], $h = 0.74$; chin: $z = 6.43$, $p < .001$, 95% CI [0.34, 0.42], $h = 0.77$; ears: $z = 8.67$, $p < .001$, 95% CI [0.43, 0.53], $h = 0.57$). In line with our number of fixations and dwell time analyses over time (Figure S3), post-hoc tests further revealed a reversal in the later fixations, showing that unexpected ROIs attracted more third or fourth fixations (expected vs. unexpected: 3rd: forehead: $z = -2.95$, $p = .01$, 95% CI [0.14, 0.23], $h = 1.23$; chin: $z = -3.33$, $p = .003$, 95% CI [0.12, 0.22], $h = 1.26$; ears: $z = -3.25$, $p = .005$, 95% CI [0.15, 0.22], $h = 1.21$; 4th: forehead: $z = -3.28$, $p = .004$, 95% CI [0.11, 0.21], $h = 1.30$; chin: $z = -3.41$, $p = .003$, 95% CI [0.10, 0.21], $h = 1.30$; ears: $z = -3.58$, $p = .001$, 95% CI [0.08, 0.20], $h = 1.34$).
